# Biofilm state *Limosilactobacillus reuteri* modulates aryl hydrocarbon receptor activity and suppresses experimental necrotizing enterocolitis

**DOI:** 10.1038/s41390-025-04351-z

**Published:** 2025-08-28

**Authors:** Nitin Sajankila, Zachary Dumbauld, Yijie Wang, Samantha J. Wala, Mecklin V. Ragan, Ameer Al-Hadidi, Samuel G. Volpe, Joseph Wickham, Lauren Mashburn-Warren, Colton Wayne, Taylor Jacobs, Siddharth Narayanan, Michael T. Bailey, Steven D. Goodman, Gail E. Besner, Belgacem Mihi

**Affiliations:** 1 Center for Perinatal Research, Nationwide Children’s Hospital, Columbus, OH USA; 2 Department of Pediatric Surgery, Nationwide Children’s Hospital, Columbus, OH USA; 3 Center for Microbial Pathogenesis, Nationwide Children’s Hospital, Columbus, OH USA; 4 Institute for Genomic Medicine, Nationwide Children’s Hospital, Columbus, OH USA

## Abstract

**Background:**

Decreased Aryl Hydrocarbon Receptor (AHR) signaling pathway activation is implicated in necrotizing enterocolitis (NEC) pathogenesis. *Limosilactobacillus reuteri* (*Lr*) is a probiotic that catabolizes tryptophan into AHR ligands. We have previously shown that *Lr* in its biofilm state has improved efficacy against NEC. However, the importance of the physiologic state of *Lr* (planktonic *vs*. biofilm) on AHR activation remains unknown.

**Methods:**

In vitro experiments using intestinal epithelial cells (IEC) and in vivo experiments in premature rodents were carried out to assess the impact of planktonic- *vs*. biofilm-state *Lr* on AHR ligand production, AHR activation, and protection against NEC.

**Results:**

Biofilm-state *Lr* was found to have increased persistence in the intestine of premature rodent pups compared to planktonic-state *Lr*. IECs exposed to conditioned media from *Lr* grown with tryptophan demonstrated increased AHR activation compared to IECs exposed to tryptophan alone. Finally, biofilm-state *Lr* was associated with increased intestinal AHR ligand production, AHR activation, and protection against NEC in rodent pups.

**Conclusion:**

Biofilm-state *Lr* has increased persistence in the gut and protects against NEC. This protection is associated with increased AHR activation in the intestine. Through improved understanding of the interactions of *Lr* and AHR signaling, we may be able to further enhance *Lr* efficacy against NEC.

**Impact:**

- *Limosilactobacillus reuteri* in its biofilm state increases AHR activation and reduces intestinal injury during NEC.
- This is the first study to look at the role of the AHR signaling pathway in *Limosilactobacillus reuteri*-mediated protection against NEC.
- Development of an effective therapy to prevent NEC would reduce the morbidity and mortality of this lethal disease.

## Introduction

Necrotizing enterocolitis (NEC) is a devastating intestinal disease of the premature neonate, with no known cure. It is characterized by exaggerated intestinal inflammation and microbial dysbiosis that can result in necrosis or perforation, with mortality exceeding 30% in the sickest neonates.^1–7^ Given the role of dysbiosis in NEC pathogenesis,^3–6^ one potential treatment strategy against the disease is the use of enteral probiotics to restore eubiosis.^8–11^ However, results in clinical trials using current formulations have been mixed.^12–14^ This lack of consistent efficacy in human neonates may be due to the physiologic state of the probiotics when they are enterally delivered.

Probiotics administered enterally encounter several obstacles that limit their ability to colonize the intestine and exert a therapeutic effect, including turbulent intraluminal forces, gastric acid, microbial competition, and the host immune system.^15,16^ One method naturally employed by bacteria to overcome these environmental factors and increase intestinal adherence is the production of a biofilm, a community of microorganisms encased in a self-made extracellular matrix composed of proteins, lipids, DNAs, and oligosaccharides.^15,17^ While production of biofilms by pathogenic bacteria can reduce clearance of these microbes and cause chronic disease, for probiotic bacteria the biofilm state can facilitate a more lasting benefit. At present, all probiotic formulations on the market and those used in clinical trials exist in their planktonic (free-living) state, rather than a biofilm state.^14^

*Lactobacillus reuteri* (*Lr*), recently reclassified as *Limosilactobacillus reuteri*,^18^ is a biofilm-producing, gram-positive bacterium with therapeutic potential against NEC.^19^ Importantly, it is naturally found in human breastmilk^20^ and can be safely administered to vulnerable neonates.^11^ We have developed a novel enteral probiotic delivery system in which *Lr* is delivered in its biofilm state by brief incubation with biocompatible porous dextranomer microspheres (DM). Importantly, DM can be loaded with beneficial substances such as maltose (DM-Malt) to further promote biofilm production and enhance *Lr* efficacy. We have previously shown that *Lr* administered in its biofilm state (*Lr*-DM-Malt) is highly effective in attenuating NEC in rats and piglets.^21–24^ In addition, in a recent Phase 1 clinical trial, we found that *Lr*-DM-Malt is also safe when administered to adult humans,^25^ paving the way for a future clinical trial in neonates. Although *Lr* in its biofilm state is beneficial in protecting the intestines from experimental NEC, the mechanisms for these effects remain to be fully elucidated.

An additional benefit of *Lr* delivery in its biofilm state may be due to enhanced adherence of the probiotic to the intestinal epithelium. The formation of a biofilm by *Lr* is dependent upon the expression of extracellular glucosyltransferase W, which acts both as an adhesin to DM as well as generates a glucan extracellular matrix derived from its substrate maltose.^24^ Moreover, *Lr* in its biofilm state (*Lr*-DM-Malt) has increased adherence to intestinal epithelial cells and mucus in vitro compared to planktonic *Lr*.^24^ However, it is not yet clear whether the state of enteral administration of *Lr* (planktonic *vs*. biofilm) also affects its persistence in the intestinal tract in vivo, and this was investigated in the current study.

A possible mechanism underlying the beneficial effects of *Lr* is its agonistic relationship with the Aryl Hydrocarbon Receptor (AHR) signaling pathway. AHR is a cytoplasmic, ligand-dependent, transcription factor known to play a role in modulating inflammation and immunity.^26^ In adults, AHR activation is important in the development of several immune cell types^27–33^ and in the maintenance of the intestinal barrier.^34–36^ Through the intracellular microbial enzyme aromatic aminotransferase (ArAT),^37^ *Lr* can metabolize dietary tryptophan (Trp) into a range of indole products that are secreted by the bacteria and can activate AHR.^38^ Recently, it was demonstrated that disruption of AHR signaling increased the susceptibility of neonatal mice to NEC, while administration of the AHR ligand indole-3-carbinol at the onset of experimental NEC attenuated intestinal injury.^39,40^ Given *Lr*’s ability to catabolize Trp into AHR ligands, and the ability of AHR ligands to alleviate NEC, it is possible that *Lr*-mediated protection against NEC may occur in part as a result of AHR activation.

Alternatively, *Lr* can influence the host’s ability to catabolize Trp into AHR ligands by increasing host Indoleamine 2,3-dioxygenase 1 (IDO1) enzyme expression, as a direct result of *Lr*’s metabolism of arginine (Arg) into ornithine.^41^ In fact, when *Lr*’s ability to produce ornithine is limited, *Lr* has reduced ability to activate AHR and protect against colitis in adult mice.^41^ Thus, it is also possible that *Lr*-mediated protection against NEC results from its interaction with host IDO1 and host Trp catabolism.

In this study, we explored the impact of planktonic-state *vs*. biofilm-state *Lr* on Trp catabolism and AHR activation during NEC. We hypothesized that the delivery of *Lr* in its biofilm state would increase persistence in the gastrointestinal tract and augment host-probiotic interactions, resulting in increased Trp catabolism into AHR ligand, increased host expression of IDO1, increased AHR activation, and protection against NEC.

## Methods

All animal protocols used were approved by the Institutional Animal Care and Use Committee (IACUC) of the Research Institute at Nationwide Children’s Hospital (protocol #AR15-00012). All work performed in this study was completed in compliance with the Guide for Care and Use of Laboratory Animals of the National Institute of Health.

### Planktonic and Biofilm *Lr* Preparation

*Lr* was prepared as we have previously described.^22–24,42–44^ In brief, 20 mg of Sephadex^TM^ G-25 Superfine DM (DM) (Cytiva, Wilmington, DE) and 80 μL 1 M maltose solution were incubated at 25 °C overnight to allow for maltose absorption and hydration of the DM (DM-Malt). Human feces-derived *Lr* 23272 (DSM 20016), obtained from ATCC and stored in 15% (v/v) glycerol, was inoculated in DeMan, Rogosa, and Sharpe (MRS) broth, and cultured for 18–20 h at 37 °C with 5% CO_2_. Following broth culture, *Lr* cells were washed in Dulbecco’s phosphate-buffered saline (PBS), and then resuspended in saline to create a fresh planktonic stock of *Lr* for each experiment. To generate *Lr*-DM-maltose, 1 mL of *Lr* stock suspension was added to the previously prepared DM-Malt and incubated at 25 °C for 30 min to allow the adhesion of *Lr* to maltose-loaded DM (DM-Malt). Serial dilutions of *Lr* stock were cultured on MRS agar for 24 h at 37 °C with 5% CO2 in order to calculate the concentration of *Lr* stock used to make planktonic *Lr* and *Lr*-DM-Malt (*Lr* in its biofilm state).

### Persistence of *Lr* in the Intestinal Tract of Unstressed Rat Pups

C-sections were performed on time-pregnant Sprague-Dawley dams (Indianapolis, IN) at E20.5 to obtain premature rat pups. On day 1 of life (DOL 1), pups were randomized to receive PBS control, 0.1 ml of planktonic *Lr* (1 × 10^8^ CFU/pup), or 0.1 ml of *Lr*-DM-Malt (1 × 10^8^ CFU/pup and 2 mg DM/pup), using a bioluminescent strain of *Lr* (LMW503).^24^ In brief, bioluminescent *Lr* was generated by transforming *Lr* 23272 using a chloramphenicol-resistant plasmid containing the click beetle luciferase gene (*CBluc*), downstream of the constitutive *Lr* elongation factor Tu promoter.^45^ Chloramphenicol (5 μg/ml) was then used to select colonies that were bioluminescent when the substrate for click beetle luciferase, D-luciferin, was added. Pups were gavage fed rat pup formula, generated by combining 15 g of powdered Similac 60/40 (Abbott Nutrition, Columbus, OH) with 75 ml of canned Esbilac (Pet-Ag, New Hampshire, IL), five times daily. Feeds were administered by orogastric gavage using a 1.9 French PICC-Nate silicone catheter (Utah Medical Products Inc, Midvale, Utah), with volumes scaled to increase daily until goal feeds (0.4 ml) were reached. Pups were housed in temperature (37 °C) and humidity-controlled Bistos baby incubators (BT-500, Medical Device Depot, Ellicott City, MD). After 72 h, rat pups received Rediject D-Luciferin (100 μl; 3 mg/pup) (Caliper Life Sciences, Hopkinton, MA) in formula by gastric gavage, and sacrificed 3 h later. The entire intestinal tract from stomach to rectum was collected *en bloc* and imaged using the bioluminescent Xenogen In Vivo Imaging System (IVIS) (PerkinElmer, Akron, OH). Data were analyzed using Living Image 4.4 software.

### Measuring the presence of AHR ligand using an AHR reporter cell line

For the purposes of these experiments, *Lr* in its biofilm state was produced by incubating *Lr* on the surface of DM that was not loaded with maltose. This was necessary to ensure that *Lr* preferentially catabolized Trp.^38^ Assessment of adherence to DM, following a previously described protocol,^24^ revealed that 39% of *Lr* was adherent to DM. To produce *Lr* supernatants, planktonic *Lr* and *Lr* in its biofilm state (*Lr*-DM) (~1 × 10^8^ CFU) were cultured in peptone-tryptone water (10 g/L tryptone, 5 g/L NaCl) supplemented with 0.6 mM L-tryptophan and incubated for 14 h at 37 °C under anaerobic conditions. Supernatants were harvested and passed through 0.2-μm filters to generate *Lr*-DM+Trp and *Lr*+Trp conditioned media. For the luciferase assays, *Lr* supernatants were added to HT-29 lucia AHR reporter cells (InvivoGen, San Diego, CA) at a final concentration of 10% in cell culture medium for 12 h. Cells cultured in culture medium containing 10% vol/vol of Peptone-tryptone water supplemented with 0.6 mM L-tryptophan were used as a negative control while cells treated 5 $\mu$M of 2-(1′H-I′-carbonyl)-thiazole-4-carboxylic acid methyl ester (ITE) were used as a positive control. Following the manufacturer’s instructions, 50 μl of QUANT-Luc™ 4 solution was added to 20 μl of cell culture medium from each culture condition. Luciferase activity was then measured using the Biotek Synergy HTX luminometer. Each condition was tested in triplicate and the experiment was repeated twice.

### Rat model of NEC and administration of *Lr*

The rat model of NEC was performed as we have previously described.^22,43^ Briefly, C-sections were performed on time-pregnant Sprague-Dawley dams (Indianapolis, IN) at E20.5. Pups were randomized to receive DM-Malt alone (2 mg DM/pup), 0.1 ml of planktonic *Lr* (1 × 10^8^ CFU/pup), or 0.1 ml of *Lr*-DM-Malt (1 × 10^8^ CFU/pup and 2 mg DM/pup). Pups were fed and housed as described above. To induce NEC, pups received lipopolysacharide (LPS; 2 mg/kg) on the first day of life (DOL 1), and were exposed to repeated episodes of hypoxia (<5% of oxygen x 90 s) and hypothermia (4 °C x 10 min) shortly after feeds three times a day. Pups were sacrificed upon reaching humane endpoint criteria (abdominal distention, cyanosis, respiratory distress, emesis), or at the end of the experiment at 96 h. Pups that died within the first 24 h of life were excluded from analysis as they were unlikely to have developed NEC this early. Rats were used in our earlier work examining intestinal indolelactate concentration after *Lr* administration during NEC, and in our studies examining persistence of *Lr* in the GI tract. We subsequently transitioned to the mouse model of NEC in our laboratory in order to facilitate future planned studies utilizing genetically altered mice. Thus, subsequent experiments in this study utilized a murine NEC model as described below.

### Intestinal indolelactate concentration in rat pups exposed to NEC

Intestinal indolelactate concentration in rat pups exposed to the rat NEC protocol was measured using the Metabolon Inc. UPLC/MS-MS platform, as previously described.^46^ Intestinal contents were harvested from the entire colon of each rat pup. Intestinal contents from the distal small intestine was not collected as the distal small intestine was preserved for histologic analysis. Raw data was then processed by Metabolon Inc. in order to generate the relative abundance of indolelactate.

### Mouse model of NEC and administration of *Lr*

The mouse NEC model we employed was adapted from a well-published model that requires repeated exposure to hypoxia and hypothermia, as well as exposure to LPS and harmful enteric bacteria with every feed.^47^ Briefly, 4-day-old C57BL/6J pups were separated from dams and housed in neonatal incubators at 37 °C for 2 h to allow for acclimation. Enteric bacteria previously obtained from the ileum of a patient with NEC requiring surgical resection (NECteria) were cultured in 20 ml of Luria-Bertani (LB) at 37 °C. After achieving an optical density at 600 nm wavelength of 0.6, corresponding to the exponential growth phase, NECteria bacteria were washed and added to 20 ml of Mouse NEC formula. The base Mouse NEC formula consisted of 2:1 Similac Advance Optigro with Iron infant formula and Esbilac canine milk replacer. In addition, LPS (2.5 mg/kg) was added to create a final Mouse NEC formula for use in experimentation. Pups received Mouse NEC formula (consisting of formula with added LPS and NECteria) six times daily via a 1.9 French PICC-Nate silicone catheter. Pups received 70 µL of formula on DOL 4, and advanced to 80 µL of formula on DOL 5-6. Pups were exposed twice daily to hypoxia (<5% oxygen x 10 min) followed by hypothermia (4 °C x 10 min). Following hypoxic/hypothermic stress, pups were returned to neonatal incubators at 37 °C. On DOL 4, pups were randomized to receive saline, 0.08 ml of planktonic *Lr* (0.8 × 10^8^ CFU/pup), or 0.08 ml of *Lr* in its biofilm state (0.8 × 10^8^ CFU/pup and 1.6 mg DM/pup). Additional pups were kept with their dams as dam-fed (DF) controls. Pups were sacrificed upon reaching humane endpoint criteria, with any remaining pups euthanized after 72 h of exposure to stress. Pups that died within the first 24 h of exposure to stress were excluded from analysis.

### Histologic assessment of terminal ileum and injury grading of mouse NEC

Following euthanasia, 2 cm of distal terminal ilium (TI) was harvested from each mouse pup. TI samples were opened longitudinally and pinned flat. Fixation was performed in 10% formalin for 24 h prior to storage in 70% ethanol. Tissue samples were embedded in agar to ensure proper orientation, followed by paraffin wax embedding.^39,47,48^ Tissue samples were sectioned via microtome (5 μm thick) and stained with Hematoxylin and Eosin (H&E) for histological analysis. H&E-stained tissue sections were assessed for the presence of intestinal injury by an investigator blinded to the study conditions. Tissue sections were imaged using a U-Tv0.5XC-3 Olympus microscope and analyzed using ImageJ software. Normal tissue sections were characterized by a high density of long villi with no visible signs of disruption or damage. Mild lesions were characterized by areas with partial villous destruction reflected by a substantial reduction in villous length and density. Severe lesions were characterized by complete villous loss and significant disruption to the mucosal architecture.^39,47,48^ The most severe lesion present in the studied tissue section was used to determine a NEC severity score for each pup. A NEC severity score of 0 was assigned to pups with no lesions, a score of 1 was assigned to pups with mild lesions, and a score of 2 was assigned to pups with severe lesions.

### mRNA expression in intestinal samples by qRT-PCR

Distal TI samples (1 cm) were flushed with PBS, frozen on dry ice, and stored at -80°C until ready for use. Samples were lysed in Trizol^TM^ reagent (Thermo Fisher Scientific) using Tissue Lyzer II (QIAGEN, Hilden, Germany). RNA extraction was performed per the manufacturer’s instructions. RNA concentration and purity were determined with a spectrophotometer (NanoDrop^TM^, Thermo Fisher Scientific). Complementary DNA (cDNA) was synthesized using the QuantiTect Reverse Transcription Kit (QIAGEN, Hilden, Germany), according to the manufacturer’s instructions. qRT-PCR using IQ SYBR Green Supermix (Thermo Fisher Scientific, Waltham, MA) and the primers described in Table 1 to quantify *Cyp1a1*, *Ido1*, and *Rplp0* mRNA expression.^39,48,49^

**Table 1:** List of qPCR primers used in this study.

Gene	Species	Forward	Reverse
*Cyp1a1*	Mouse	3’-GACCCTTACAAGTATTTGGTCGT-5’	5’-GGTATCCAGAGCCAGTAACCT-3’
*Ido1*	Mouse	3’-CGGACTGAGAGGACACAGGTTAC-5’	5’-ACACATACGCCATGGTGATGTAC-3’
*Rplp0*	Mouse	3’-GGCGACCTGGAAGTCCAACT-5’	5’-CCATCAGCACCACAGCCTTC-3’

### IDO1 Immunohistochemistry (IHC) of intestinal samples

IHC of mouse pup terminal ileum (distal 2 cm) was performed using a method we have previously described.^21^ Briefly, tissue samples were fixed in 10% neutral buffered formalin for 24 h at room temperature (RT). Paraffin-embedded tissues were sectioned at 5 μm thickness, deparaffinized in xylene, and rehydrated in ethanol. Antigen retrieval was performed by incubating slides in 10 mM sodium citrate buffer (pH 6.0) in a boiling water bath for 40 min. Endogenous peroxidase was quenched by incubating with 0.3% hydrogen peroxide in methanol for 30 min. Tissue sections were incubated in blocking buffer containing 1% bovine serum albumin (BSA), 2% goat serum, 0.1% Triton, and 0.05% Tween 20 for 2 h at RT. The primary antibody used was an anti-IDO1 polyclonal antibody (BS-7659R; Bioss Antibodies, Woburn, MA) at a 1:150 dilution. Samples were incubated with the primary antibody at 4 °C overnight. The following day, samples were incubated with 1:500 biotinylated anti-rabbit IgG secondary antibody (111-065-046; Jackson ImmunoResearch, West Grove, PA) at RT. Samples were then treated with Avidin–Biotin Complex (ABC) horseradish peroxidase (HRP) reagents (Vector Lab, Newark, CA) for 30 min at RT and visualized by incubation with 3,3’-Diaminobenzidine tetrahydrochloride (DAB) (Sigma, D5905, Burlington, MA), followed by mounting with DPX solution (Sigma, Burlington, MA). Images were obtained using an ECLIPSE Ti2 inverted microscope, equipped with the NIS-Elements workstation, at 20x magnification (Nikon, Tokyo, Japan). A semi-quantitative analysis was performed on the processed images using ImageJ (U.S. National Institutes of Health, Bethesda, Maryland), following a previously published protocol developed by Crowe and Yue.^50^ IDO1 staining was normalized to the number of nuclei, allowing for fair quantification of IDO1 staining between groups.

### Statistical analyses

All statistical analyses were performed using GraphPad Prism version 9 for Windows (GraphPad Software, San Diego, California). Simple comparisons of means, including severity of NEC or changes in relative expression of inflammatory markers, were compared using an unpaired student’s *t*-test. Comparison of multiple means was performed using an ordinary one-way ANOVA, and unless specified multiple comparisons were performed using Fisher’s least square difference (LSD). For measuring intestinal indolelactate concentration, equal standard deviations were not assumed, and multiple comparisons were performed using unpaired t-tests with Welch’s correction. A *p*-value of <0.05 was considered to be statistically significant.

## Results

### Persistence of *Lr* in the intestines of premature rat pups is increased by delivery in its biofilm state

**Fig. 1:**
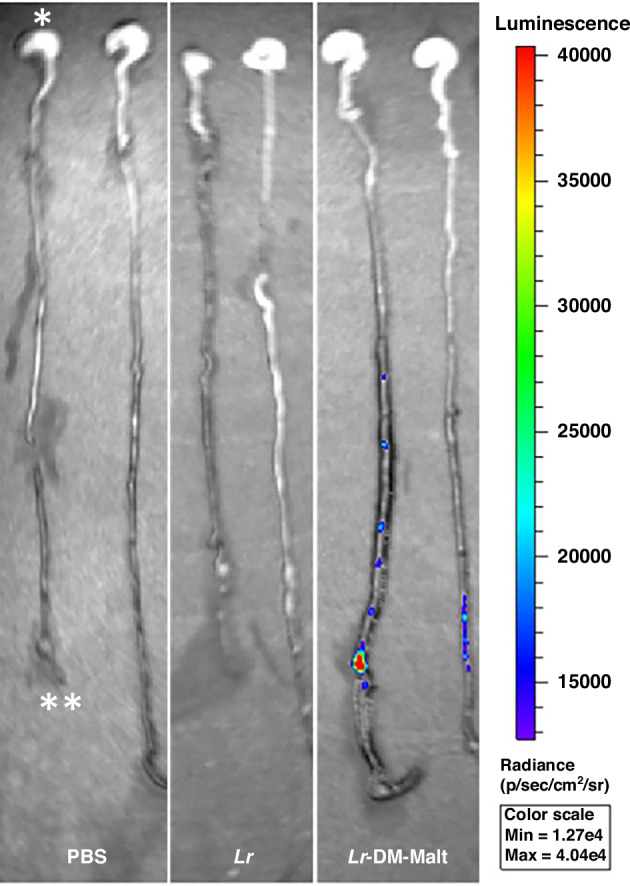
*Lr* administered in its biofilm state has increased persistence in the intestines of premature rat pups. Rat pups were delivered by C-section and treated on DOL-1 with PBS, bioluminescent *Lr* in its planktonic state (*Lr*), or bioluminescent *Lr* in its biofilm state (*Lr*-DM-Malt). After 72 h, pups received 100 μl (3 mg/pup) of Rediject D-Luciferin in formula via gastric gavage, and were sacrificed 3 h later. The entire intestinal tract from the stomach (*) to the rectum (**) was collected in one piece and then imaged using Xenogen IVIS and analyzed using Living Image 4.4 software. Bioluminescent *Lr* was only visualized in the intestinal tract of pups that received *Lr* in its biofilm state (*Lr*-DM-Malt), with *Lr* identified throughout the distal small intestine and colon.

Premature unstressed rat pups delivered by C-section (E20.5) received a single dose of PBS, bioluminescent *Lr* in its planktonic state (*Lr*), or bioluminescent *Lr* in its biolfilm state (*Lr*-DM-Malt) on DOL 1, with persistence of *Lr* in the intestine assessed after 72 h. The 72 h timepoint was selected for study as this is the point at which maximal death secondary to NEC occurs in our experimental NEC models. Bioluminescent *Lr* was identified in the intestines of pups that received *Lr*-DM-Malt but not pups receiving planktonic *Lr* (Fig. 1), demonstrating that when delivered in its biofilm state, *Lr* exhibited enhanced persistence in the gastrointestinal (GI) tract.

### Biofilm formation does not affect *Lr’s* ability to catabolize Trp into AHR ligands

**Fig. 2:**
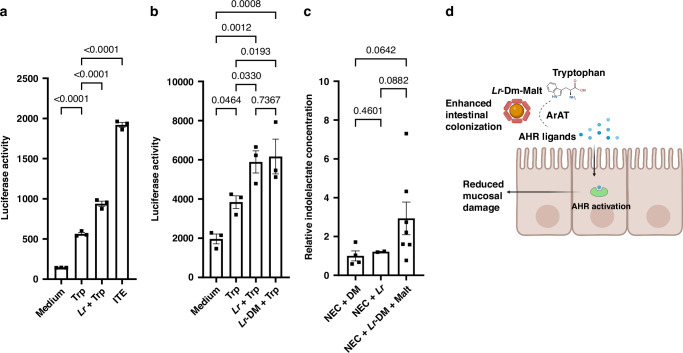
*Lr* catabolizes dietary tryptophan and increases indolelactate, an AHR ligand. **a** In vitro assay examining the ability of *Lr* grown in the presence of tryptophan to produce AHR ligand. HT29-Lucia AHR reporter cells were grown to confluence and then exposed to culture medium, culture medium containing tryptophan (Trp), conditioned media from planktonic *Lr* grown in the presence of tryptophan (*Lr*+Trp), or ITE, a potent AHR activator. Luciferase activity was measured using the Biotek Synergy HTX luminometer. Data were analyzed using an ordinary one-way ANOVA, and multiple comparisons were performed using Fisher’s LSD. Compared to tryptophan alone, conditioned media from *Lr* grown in the presence of tryptophan led to increased luciferase activity (AHR activation). This shows that human *Lr 23272* is capable of catabolizing tryptophan into an AHR ligand. **b** HT29-Lucia AHR reporter cells were grown to confluence and then exposed to culture medium, culture medium containing 10% vol/vol of Peptone-tryptone water supplemented with 0.6 mM L-tryptophan (Trp), conditioned media from planktonic *Lr* grown in the presence of tryptophan (*Lr*+Trp), and conditioned media from biofilm state *Lr* grown in the presence of tryptophan (*Lr*-DM+Trp). Luciferase activity (AHR activation) was measured as described above. No differences were observed in AHR activation between cells treated with *Lr*+Trp and *Lr*-DM+Trp. This suggests that the biofilm state does not directly result in an increase in AHR activation by *Lr*. **c** In vivo study examining the effect of state of delivery of *Lr* (planktonic *vs*. biofilm) on levels of indolelactate, an AHR ligand. Rat pups delivered by C-section at E20.5 were treated on DOL 1 with DM+Malt (no *Lr*), planktonic *Lr* (no DM-Malt), or *Lr* in its biofilm state (*Lr*-DM-Malt) and exposed to our experimental rat NEC protocol. Intraluminal indolelactate concentration was measured using the Metabolon Inc. UPLC/MS-MS platform. Indolelactate concentration was analyzed using an ordinary one-way ANOVA; equal standard deviations were not assumed, and multiple comparisons were performed using unpaired t-tests with Welch’s correction. Pups exposed to NEC that were treated with *Lr*-DM-Malt had the greatest intraluminal concentration of indolelactate, suggesting improved catabolism of dietary tryptophan in the presence of *Lr* in its biofilm state. In these experiments, a *p*-value of <0.05 was considered to be statistically significant. Data represent mean ± standard error. **d** Schematic demonstrating the proposed mechanism through which *Lr*, in its biofilm state, increases AHR activation. Enterally administered *Lr* in its biofilm state has improved colonization in the intestine. Upon intestinal colonization, *Lr* converts dietary Trp into AHR ligands, resulting in increased intestinal AHR activation and protection of the intestinal mucosa against inflammatory insults.

Previous studies have demonstrated that different murine strains of *Lr* have the ability to convert Trp into a range of AHR ligands, including indole-3-lactate (indolelactate), indole-3-acetate, indole-3-glyoxylicacid, and indole-3-aldehyde, that can activate AHR in the host.^33,51^ Cervantes-Barragan et al. showed that *Lr* lacking *Arat*, a key enzyme in Trp catabolism in AHR ligands, results in abrogation of AHR activation.^33^ To test whether human *Lr* 23272 (DSM 20016) is also able to produce AHR ligands when Trp is present, we used an AHR cell reporter line. Colonic HT-29 Lucia AHR reporter cells were exposed to cell culture medium alone (negative control), Trp (0.6 mM) (diluted 10% vol/vol in culture medium), conditioned media from planktonic *Lr* (~1 × 10^8^ CFU) + Trp (0.6 mM) (diluted 10% vol/vol in culture medium), or ITE (5 $\mu$M), a potent AHR ligand (positive control). As expected, the presence of Trp in the culture medium resulted in AHR activation through colonic cell catabolism of Trp into AHR ligand, measured by an increase in luciferase activity by the reporter cell line (Fig. 2a). Conditioned media from *Lr* grown in a planktonic state in the presence of an equal concentration of Trp, was found to significantly increase AHR activation compared to cells exposed to Trp alone, (Fig. 2a). This confirms that human *Lr* 23272, as previously demonstrated in murine strains,^33,51^ can produce AHR ligands in the presence of Trp.

We subsequently compared conditioned media from either *Lr* grown in a planktonic state (as specified above) or *Lr* grown in a biofilm state on the surface of DM, *Lr* (~1 × 10^8^ CFU)-DM (2 mg/mL), in the presence of an equal concentration of Trp. Interestingly, we found no significant difference in AHR activation between cells treated with supernatants from *Lr* grown in its planktonic state and those treated with supernatant from *Lr* grown in its biofilm state, in the presence of Trp (Fig. 2b). This suggests that the biofilm state does not directly increase the ability of *Lr* to metabolize Trp into AHR ligands.

We next sought to examine the impact of delivering *Lr* in its planktonic *vs*. biofilm state on intestinal AHR ligand levels during experimental NEC. It has previously been demonstrated that indolelactate, an AHR ligand produced through *Lr*’s ArAT enzyme, is important in the AHR-dependent induction of gut intraepithelial CD4 + CD8$\alpha \alpha \,$+T cells by *Lr* in adult mice.^33^ To assess the impact of *Lr* administration on intestinal indolelactate on NEC severity, rat pups delivered by C-section (E20.5) and exposed to the experimental NEC protocol were treated with DM-Malt, planktonic *Lr*, or *Lr* in its biofilm state (*Lr-*DM-Malt). Levels of intestinal indolelactate were measured. There was no difference in intestinal indolelactate levels in pups treated with either DM-Malt alone (no *Lr*) or planktonic *Lr* alone (no DM-Malt) (Fig. 2c). In contrast, pups treated with *Lr* in its biofilm state (*Lr-*DM-Malt) had greater intestinal indolactate levels than either planktonic *Lr* or DM-Malt alone, approaching significance (Fig. 2c). Thus, delivery of *Lr* in its biofilm state increased catabolism of intestinal Trp into the AHR ligand Indolelactate. Taken together, these data suggest that the increased concentration of AHR ligands in the intestine of pups that received *Lr*-DM-Malt compared to planktonic *Lr* is likely to be due to improved colonization when the probiotic is delivered in its biofilm state, rather than an increased ability to catabolize Trp into AHR ligands. It is ultimately the AHR ligands that confer protection of the intestinal mucosa against inflammatory insults during NEC (Fig. 2d).

### Protection against NEC by *Lr* in its biofilm state is associated with increased intestinal AHR activation

**Fig. 3:**
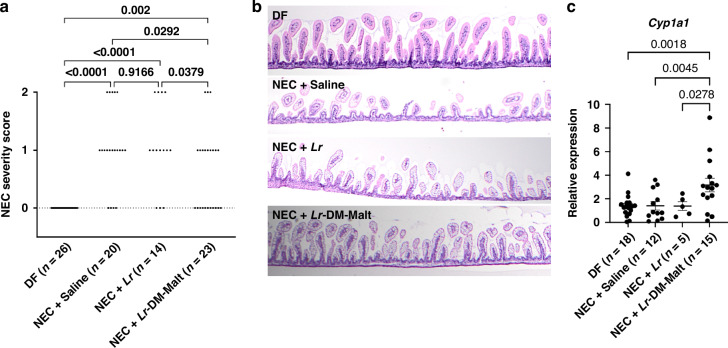
Protection against NEC by *Lr* in its biofilm state is associated with increased AHR activation. **a** NEC Severity Scores. Mouse pups were treated on DOL 3 with saline, planktonic *Lr*, or *Lr* in its biofilm state (*Lr*-DM-Malt) and exposed to our experimental mouse NEC protocol. Terminal ileum was collected from pups exposed to NEC and the severity of NEC was scored on a scale of 0 to 2, where 0 represented no disease, 1 represented mild disease, and 2 represented severe disease. Treatment with *Lr-*DM-Malt significantly reduced the severity of NEC compared to pups treated with saline or planktonic *Lr*. **b** Representative histology from pups exposed to NEC that were treated with saline, planktonic *Lr*, or *Lr* in its biofilm state (*Lr*-DM-Malt). Pups treated with *Lr* (planktonic or biofilm) had improved preservation of villi compared to pups that received saline. Compared to pups treated with planktonic *Lr*, pups treated with *Lr* in its biofilm state also had improved density of villi. **c** AHR activation measured by *Cyp1a1* gene expression via qRT-PCR. In mouse pups exposed to experimental NEC, treatment with *Lr-*DM-Malt significantly increased *Cyp1a1* expression (AHR activation) compared to administration of saline or planktonic *Lr*. Comparison of multiple means, for both histology and gene expression, was performed using an ordinary one-way ANOVA, and multiple comparisons were performed using Fisher’s LSD. A *p*-value of <0.05 was considered to be statistically significant. Data represent mean ± standard error.

It has previously been demonstrated that oral administration of indole-3-carbinol, another AHR ligand, to mice exposed to experimental NEC led to reduced intestinal inflammation and NEC severity.^39^ After showing that treatment with *Lr* in its biofilm form led to an increase in aryl hydrocarbon receptor (AHR) ligand production, we further investigated whether delivering *Lr* in vivo in its biofilm state could augment AHR activation and protection against NEC. This was achieved using a well-established mouse model of NEC.^39,47,48^ Mouse pups exposed to experimental NEC received saline control, planktonic *Lr*, or *Lr* in its biofilm state (*Lr*-DM-Malt). Additional control pups were dam fed and unstressed (DF). No difference in survival was found between the experimental groups (Supplementary Fig. S1). However, pups that were subjected to experimental NEC and treated with *Lr*-DM-Malt, had significantly improved NEC severity scores compared to pups that received either saline control or planktonic *Lr* during experimental NEC (*p* < 0.05 compared to either saline or planktonic *Lr*) (Fig. 3a). Representative histology from pups exposed to NEC that were treated with saline, planktonic *Lr*, or *Lr* in its biofilm state (*Lr*-DM-Malt) is displayed in Fig. 3b. Pups treated with *Lr* in its biofilm state had improved preservation of villous arhictecture and density compared to either saline or planktonic *Lr* (Fig. 3b). In addition, we used qRT-PCR to measure gene expression of *Cyp1a1*, which is an AHR-dependent gene that has been previously used by others as a readout for AHR activation.^39,40^ The improved protection by *Lr* in its biofilm state was associated with a significant increase in *Cyp1a1* gene expression (indicative of increased AHR activation) compared to treatment with saline or planktonic *Lr* (p < 0.05) (Fig. 3c).

### Protection against NEC by *Lr* in its Biofilm State is Associated with Increased Expression of host *Ido1*

**Fig. 4:**
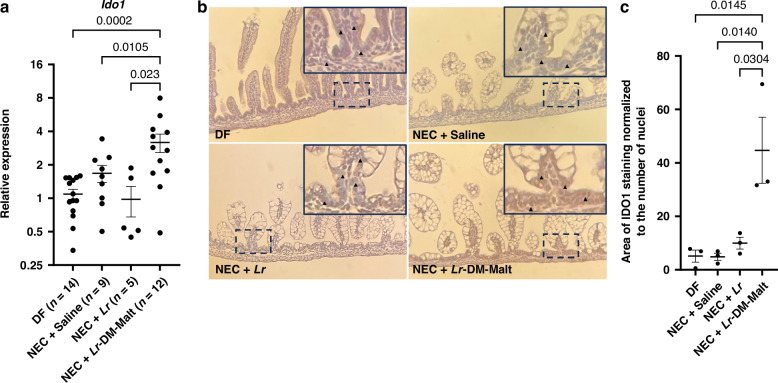
*Ido1* gene expression is increased in pups exposed to NEC and treated with *Lr* in its biofilm State. **a** *Ido1* gene expression via qRT-PCR. Mouse pups were exposed to experimental NEC and treated as indicated. Treatment with *Lr-*DM-Malt significantly increased *Ido1* gene expression compared to either saline or planktonic *Lr*. **b** IDO1 immunohistochemistry. Mouse pups were exposed to experimental NEC and treated as indicated. Intestinal tissue sections were stained with rabbit anti-IDO1 polyclonal antibody, followed by conjugation to biotinylated anti-rabbit IgG secondary antibody and DAB staining. Images displayed were taken using a 10x objective. For each condition, representative areas (indicated by dotted squares) were digitally magnified (upper right corner insets). IDO1 staining intensity was observed in the NEC + *Lr*-DM-Malt group but not in the other groups. Relevant areas with differential IDO1 staining intensity are indicated by arrowheads. **c** Semi-quantitative analysis of IDO1 immunohistochemistry. Images were processed and analyzed using ImageJ, following a previously published protocol. For each image, the area of IDO1 staining was normalized to the number of nuclei, allowing for fair quantification of IDO1 staining between groups. A significant increase in IDO1 staining was found in pups exposed to experimental NEC and treated with *Lr*-DM-malt, compared to those treated with either saline or planktonic *Lr*. Comparison of *Ido1* gene expression and relative IDO1 immunohistochemical staining was analyzed using an ordinary one-way ANOVA, and multiple comparisons were performed using Fisher’s LSD. A *p*-value of <0.05 was considered to be statistically significant. Data represent mean ± standard error.

In addition to *Lr* metabolism of Trp into AHR ligands, the host intestine can also produce AHR ligands by Trp catabolism via the enzyme Indoleamine 2,3-dioxygenase 1 (IDO1). *Lr* catabolism of dietary arginine into ornithine has been shown to increase host IDO1, ultimately leading to increased production of host-derived AHR ligands.^41^ Given that *Lr* in its biofilm state is associated with improved persistence within the intestine, we further hypothesized that *Lr* in its biofilm state would increase host catabolism of tryptophan via IDO1. To test this hypothesis, we examined *Ido1* gene expression in the intestine of mouse pups subjected to NEC and treated with either planktonic *Lr* or *Lr* in its biofilm state (*Lr*-DM-Malt). *Ido1* expression was significantly increased in pups that were exposed to NEC and treated with *Lr*-DM-Malt, but not in those treated with planktonic *Lr* (Fig. 4a). These findings were confirmed with IDO1 immunohistochemistry (IHC) of mouse pup intestinal tissue sections (Fig. 4b). Semi-quantitative analysis revealed increased IDO1 protein staining in the intestinal epithelium of pups exposed to NEC and treated with *Lr* in its biofilm state, compared to any of the other groups (Fig. 4c). This highlights the improved ability of *Lr* in its biofilm state to beneficially interact with the host intestinal epithelium to increase intestinal AHR ligand levels.

## Discussion

NEC is the most common surgical emergency in preterm infants.^7^ Despite decades of research, there are currently no FDA-approved therapies for the disease. Bowel rest, gastric decompression, broad-spectrum antibiotics, and IV nutrition remain the primary tools to limit disease progression. However, a significant proportion of infants will still require surgery, with mortality greater than 30% in these sickest neonates.^2,52,53^ In addition, babies that survive suffer from chronic, debilitating morbidities including short-gut syndrome, cholestatic liver disease, and developmental delays.^7^ Thus, there is an urgent need for the development of better therapeutic strategies to reduce the severity and incidence of the disease. Given that intestinal dysbiosis is a predisposing factor for the development of NEC,^54,55^ one promising preventative strategy is the use of probiotics such as *Lr*.^14^

Enterally-administered probiotics encounter several environmental barriers that can decrease their efficacy.^15,16^ These environmental factors can reduce the effective dose of probiotics that reaches and beneficially modulates the target intestine. The current study demonstrates that *Lr* delivered in its biofilm state has increased persistence in the gastrointestinal tract compared to planktonic *Lr*. In addition, *Lr* administered in its biofilm state is associated with increased AHR activation and improved protection against NEC. Since *Lr* in its biofilm state does not lead to increased AHR activation in vitro, it appears that the biofilm state enhances AHR activity during NEC by facilitating improved intestinal colonization.

A key finding in the current study is that *Lr* in its biofilm state increases AHR activation, which has been shown to suppress the development of NEC in rodent models. We demonstrated that *Lr* in its biofilm state increased levels of intestinal indolelactate, which is produced through catabolism of Trp, and increased activation of AHR in the intestinal epithelium. In addition, we found that *Lr* administered in its biofilm state increased the expression of IDO1 on the intestinal epithelium. It was previously demonstrated that *Lr* can metabolize arginine into ornithine, with the resultant ornithine being able to increase intestinal *Ido1* expression,^41^ likely explaining the findings in the current study. Further work is needed to understand the importance of arginine catabolism by *Lr* in the context of NEC, and to understand the importance of host *vs*. microbial Trp catabolism into AHR ligands during *Lr*-mediated protection against NEC.

At present, limited well-controlled clinical trials of *Lr* in neonates exist. In one of the largest studies to date and with the longest duration of follow up, no impact on NEC was seen as a result of *Lr* DSM 17938 administration, despite daily enteral supplementation of human breast milk with the probiotic (1.25 × 10^8^ CFU) from DOL 3 to post-menstrual week 36.^11^ A recent systematic review of *Lr* DSM 17938 given orally to preterm infants demonstrated that administration of *Lr* was associated with a reduced time to first full feed, length of stay, and NEC, but reductions in mortality were not statistically significant.^56^ However, the certainty of the evidence regarding the impact of *Lr* on NEC was deemed to be “very low” by the authors of this meta-analysis. The lack of consistent efficacy of *Lr* against NEC in neonates, highlighted by these studies, may be related to the planktonic state in which the probiotic was delivered in all clinical trials to date.^14^

Another hurdle preventing widespread usage of probiotics such as *Lr* in Neonatal Intensive Care Units (NICU) is the unavailability of a safe product made with Good Manufacturing Practices (GMP).^14^ The American Association of Pediatrics does not recommend the routine use of probiotics in at-risk neonates.^12^ Although the routine use of probiotics in premature babies in neonatal intensive care units NICUs in the US had increased to ~30% in 2023, an abrupt decline followed after September 2023, when the FDA issued warnings that probiotic administration to prevent NEC should only utilize preparations that were FDA approved, of which none presently exist.^13^

In conclusion, delivery of *Lr* in its biofilm state, by adherence to the probiotic vehicle DM-Malt, enhances *Lr*-mediated activation of AHR in the intestine and improves protection against NEC. Future clinical trials that utilize GMP-grade *Lr* to protect neonates from NEC may benefit from the delivery of the probiotic in its biofilm state.

## Supplementary information

Supplementary figure

## Data Availability

The datasets generated during and/or analysed during the current study are available from the corresponding author on reasonable request.
