# Supplementary material for: Biofilm state Limosilactobacillus reuteri modulates aryl hydrocarbon receptor activity and suppresses experimental necrotizing enterocolitis
Source: Pediatr Res. 2025 Aug 28;99(3):1137–44. doi: 10.1038/s41390-025-04351-z (PMC13021507; doi:10.1038/s41390-025-04351-z)
Supplement: Supplementary file 1 — Supplementary figure [file 41390_2025_4351_MOESM1_ESM.pdf]

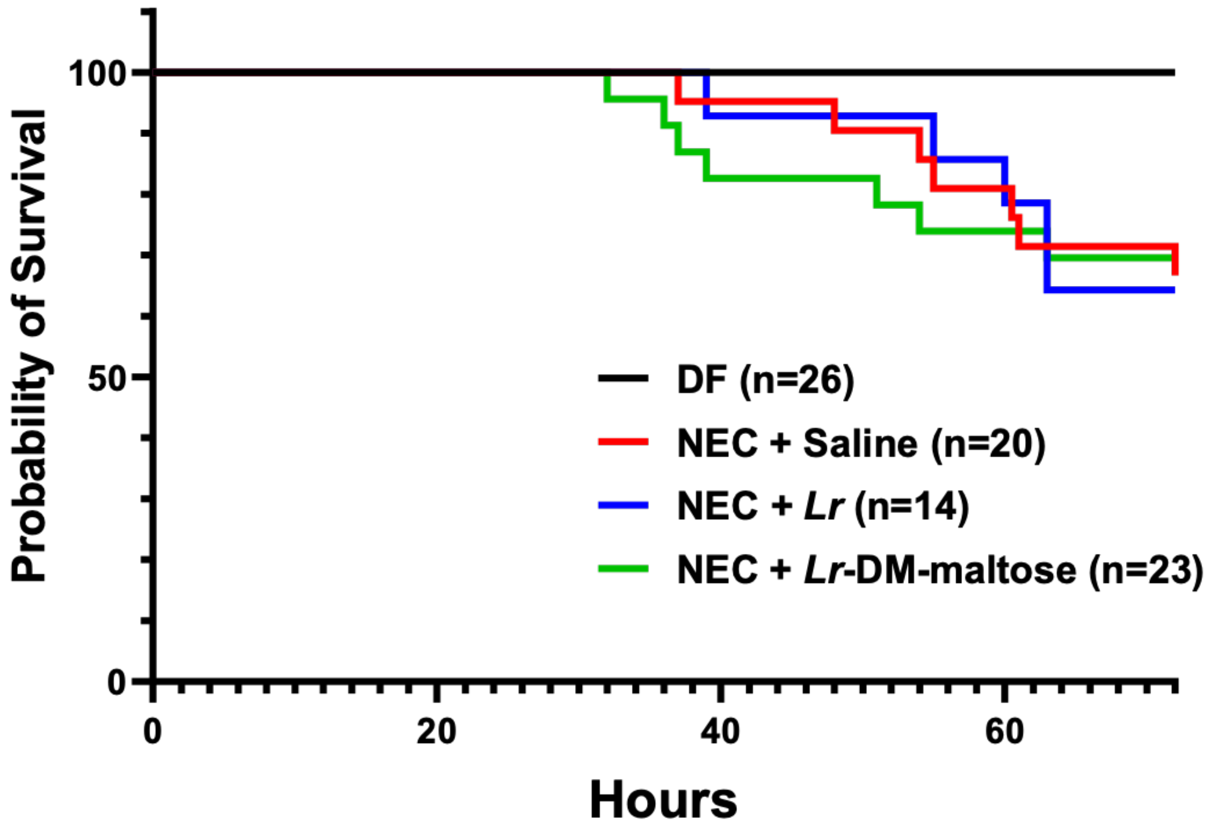


**Supplemental Figure 1. *Lr* in its biofilm state does not protect against death from experimental NEC.** Mouse pups were treated on DOL 3 with saline, planktonic *Lr,* or *Lr* in its biofilm state (*Lr*-DM-Malt) and exposed to our experimental mouse NEC protocol. Pups were sacrificed upon reaching humane endpoints or at the completion of the experiment at 72 hours. Survival analysis revealed that there was no significant difference in the probability of survival between any of the experimental groups, regardless of treatment with *Lr*.
